# Roles of N^6^‐methyladenosine (m^6^A) RNA modifications in urological cancers

**DOI:** 10.1111/jcmm.15750

**Published:** 2020-08-17

**Authors:** Xiao Wang, Haiyun Xie, Yufan Ying, Danni Chen, Jiangfeng Li

**Affiliations:** ^1^ Department of Urology First Affiliated Hospital Zhejiang University School of Medicine Hangzhou China; ^2^ Department of Radiation Oncology First Affiliated Hospital Zhejiang University School of Medicine Hangzhou China

**Keywords:** epigenetics, m^6^A, RNA modification, urological cancers

## Abstract

Epigenetics has long been a hot topic in the field of scientific research. The scope of epigenetics usually includes chromatin remodelling, DNA methylation, histone modifications, non‐coding RNAs and RNA modifications. In recent years, RNA modifications have emerged as important regulators in a variety of physiological processes and in disease progression, especially in human cancers. Among the various RNA modifications, m^6^A is the most common. The function of m^6^A modifications is mainly regulated by 3 types of proteins: m^6^A methyltransferases (writers), m^6^A demethylases (erasers) and m^6^A‐binding proteins (readers). In this review, we focus on RNA m^6^A modification and its relationship with urological cancers, particularly focusing on its roles and potential clinical applications.

## 1 INTRODUCTION

Epigenetics is commonly defined as the study of reversible and heritable phenotype alterations that do not involve DNA sequence changes.^1^ The scope of epigenetics usually includes chromatin remodelling, DNA methylation, histone modifications, non‐coding RNAs and RNA modifications. Epigenetics is well studied in cancer. In our previous studies, we elucidated that both DNA methylation and non‐coding RNAs are involved in the carcinogenesis of urological cancers (prostate cancer, bladder cancer and renal cell carcinoma).^2^, ^3^, ^4^ In recent years, RNA modifications have emerged as important regulators in a variety of physiological processes and disease progression, especially in human cancers.^5^ Common RNA modifications include 5‐methylcytosine (m^5^C), N6‐methyladenosine (m^6^A) and N7‐methylguanosine (m^7^G).^6^

Among the various RNA modifications, m^6^A is the most common.^7^ Recent studies have illustrated that m^6^A is involved in many cellular processes, including human cancers.^8^, ^9^, ^10^, ^11^ In this review, we focus on the relationship between RNA m^6^A modification and urological cancers, especially their roles and potential clinical applications.

## 2 RNA m^6^A MODIFICATION

Transcriptome‐wide analysis revealed that m^6^A modification may affect 7676 mammalian genes.^12^ Most m^6^A modification sites are located in the 3′‐untranslated regions (3′‐UTRs) and stop codons, presenting as a consensus sequence of RRACH (R = G or A; H = A, C, or U).^9^ The function of m^6^A modifications is mainly regulated by three types of proteins, writers, erasers and readers. Writers are m^6^A methyltransferases, including METTL3, METTL14, WTAP, METTL16, VIRMA, RBM15 and ZC3H13.^13^ Erasers are m^6^A demethylases, including FTO and ALKBH5.^14^, ^15^ Readers are m^6^A‐binding proteins, including YTHDFs, YTHDCs, IGF2BPs, HNRNPA2B1 and EIF3.^6^, ^16^, ^17^, ^18^, ^19^, ^20^, ^21^, ^22^, ^23^, ^24^, ^25^ The molecular structure and potential function of m^6^A regulators have been described in detail in previous reviews.^11^, ^26^, ^27^, ^28^ In brief, m^6^A methyltransferases and demethylases alter the m^6^A modification of target RNA and further recruited m^6^A‐binding proteins to determine the fate of target RNA. These m^6^A modified RNAs may further play important roles in biological processes.

## 3 ROLES OF RNA m^6^A MODIFICATION IN UROLOGICAL CANCERS

Emerging evidence shows that RNA m^6^A methylation is closely associated with the progression of urological cancers, including their carcinogenesis, proliferation and metastasis. Herein, we briefly review recent studies of m^6^A methylation in urological cancers (Table 1).

**TABLE 1:** The roles of RNA m^6^A in urological cancers

Cancer type	M6A regulators	Roles in cancer	Biological function	Mechanisms
Prostate cancer	YTHDF2^29^	Oncogene	Promoting proliferation and metastasis	Regulated by miR‐493‐3p
	METTL3^30^	Oncogene	Promoting proliferation	Regulating Hedgehog pathway
Bladder cancer	METTL3^31^	Oncogene	Promoting proliferation and metastasis	Regulating via AFF4/NF‐κB/MYC signalling network in m^6^A‐dependent way
	METTL3/YTHDF2^32^	Oncogene	Promoting proliferation and metastasis	Inhibiting the expression of SETD7 and KLF4 in m^6^A‐YTHDF2‐dependent way
	METTL3^33^	Oncogene	A prognostic indicator	—
	METTL3^34^	Oncogene	Promoting malignant transformation	Regulating via METTL3‐YTHDF1‐CDCP1 axis
	METTL3^35^	Oncogene	Promoting proliferation and progression	Regulating via METTL3‐YTHDF1/3‐ITGA6 axis
	METTL3^36^	Oncogene	Promoting carcinogenesis	Regulating pri‐miR221/222 process in m^6^A‐dependent way
	METTL3^37^	Oncogene	Biomarker	—
Renal cell carcinoma	METTL3^38^	Tumour suppressing gene	Inhibiting proliferation and metastasis/biomarker	Regulating via EMT and PI3K‐Akt‐mTOR pathways
	METTL14^39^	Tumour suppressing gene	Biomarker	Regulating PTEN
	METTL14^40^	Tumour suppressing gene	Inhibiting metastasis	P2RX6 activation promoted metastasis via ATP‐induced Ca^2+^ influx modulating ERK1/2 phosphorylation and MMP9 pathway.
	METTL3/METTL14^41^	Tumour suppressing gene	Biomarkers	Regulating of mTOR pathway
	METTL3/METTL14^42^	Tumour suppressing gene	Biomarkers	—
	FTO/ALKBH5^43^	Tumour suppressing gene	Biomarkers	—
	FTO^44^	Tumour suppressing gene	Suppressing carcinogenesis	Regulating via FTO‐PGC‐1α signalling axis

### 3.1 Prostate cancer

**FIGURE 1:**
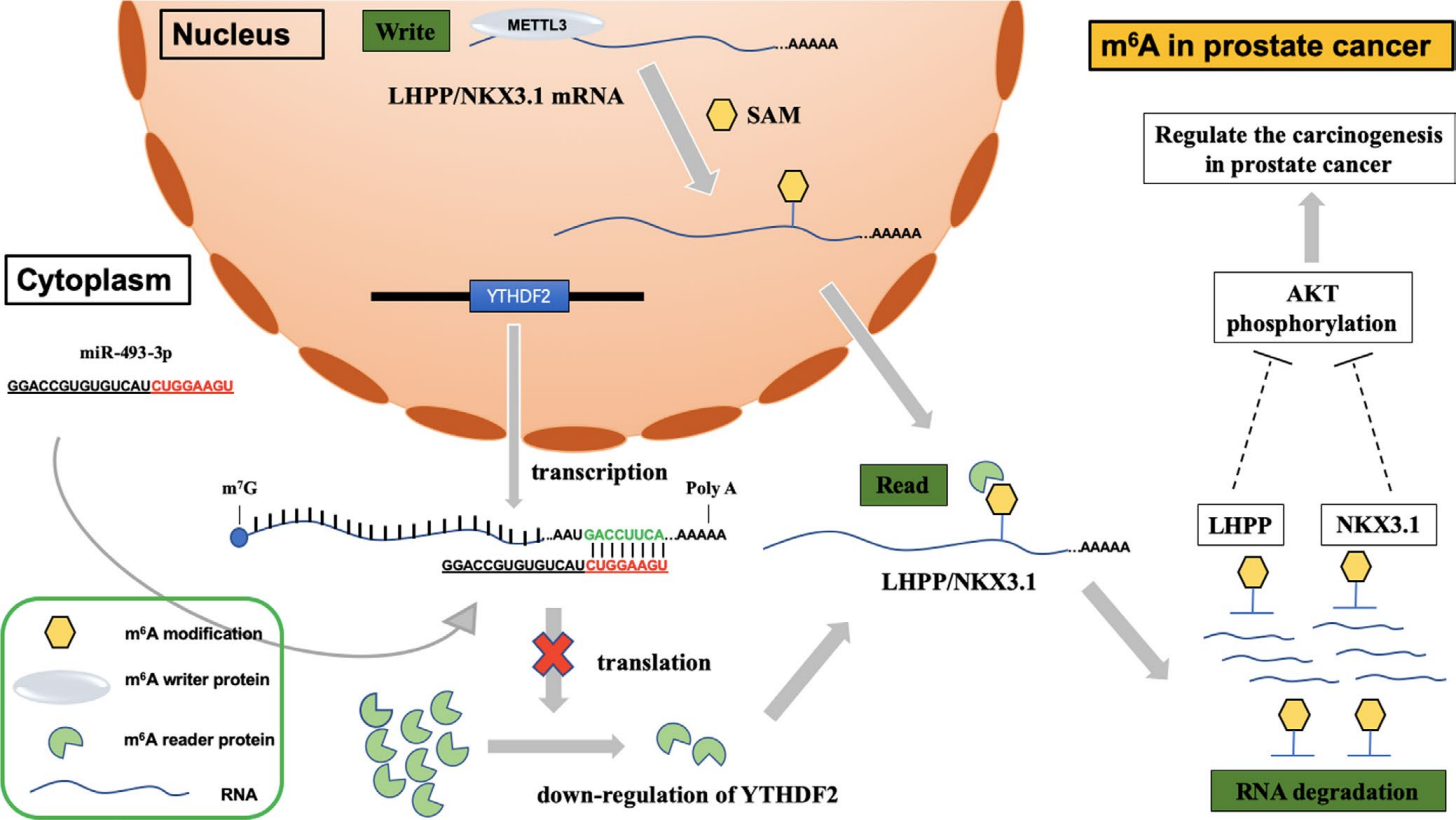
The possible mechanism of m^6^A methylation in prostate cancer. miR‐493‐3p increased the m^6^A level and inhibited tumour carcinogenesis by down‐regulating its downstream target YTHDF2 in prostate cancer, and METTL3 inhibited the expression of LHPP and NKX3‐1 in an m^6^A‐YTHDF2‐dependent manner to further promote AKT phosphorylation‐induced tumour progression in prostate cancer

Prostate cancer is one of the most commonly diagnosed cancers worldwide and is especially prevalent in developed countries.^45^ In our previous study, we found that the expression of YTHDF2 in prostate cancer was up‐regulated, and the increased expression of YTHDF2 was related to prostate cancer proliferation and metastasis.^29^ The up‐regulation of YTHDF2 in prostate cancer was possibly contributed to the regulation of miR‐493‐3p, which increased the m^6^A level and inhibited tumour carcinogenesis by down‐regulating its downstream target YTHDF2 in prostate cancer. In our ongoing study, we found that METTL3 in prostate cancer was up‐regulated and contributed to the carcinogenesis of prostate cancer. METTL3 inhibited the expression of LHPP and NKX3‐1 in an m^6^A‐YTHDF2‐dependent manner to further promote AKT phosphorylation‐induced tumour progression in prostate cancer (Figure 1). Cai et al^30^ also demonstrated that the METTL3 promoted proliferation and metastasis of prostate cancer. They further demonstrated that METTL3 regulated m^6^A modification and GLI1 expression, an important component of the hedgehog pathway.

### 3.2 Bladder cancer

**FIGURE 2:**
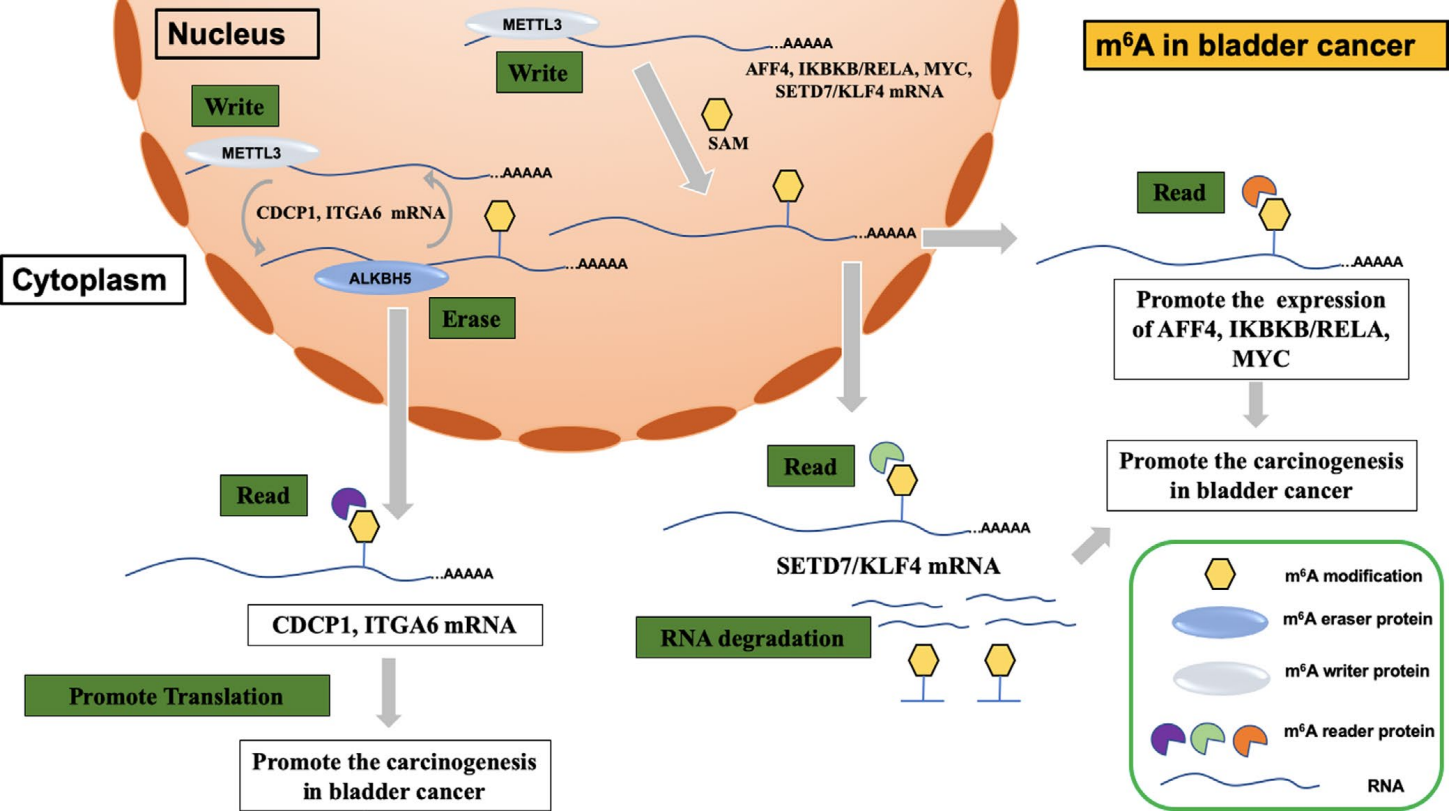
The possible mechanism of m^6^A methylation in bladder cancer. METTL3 promoted the AFF4/NF‐κB/MYC signalling network, the translation of CDCP1 and ITGA6, and inhibited the expression of SETD7 and KLF4 in an m^6^A‐dependent manner to further promote the carcinogenesis in bladder cancer. Different readers functioned differently and played crucial roles in bladder cancer (YTHDF1/2/3)

Bladder cancer is one of most commonly diagnosed cancers worldwide, especially in men.^46^ Both the incidence and mortality of bladder cancer have increased rapidly in recent years.^47^ The m^6^A methyltransferase METTL3 seems to play important roles in the carcinogenesis of bladder cancer. The role of METTL3 in bladder cancer has been exclusively studied in many research centres. Cheng et al^31^ found that the expression of METTL3 was elevated in bladder cancer and further identified AF4/FMR2 family member 4 (AFF4), key regulators of the NF‐κB pathway (IKBKB and RELA) and MYC as direct downstream targets of METTL3. METTL3 promoted the proliferation and metastasis of bladder cancer via the AFF4/NF‐κB/MYC signalling network in an m^6^A‐dependent manner. Similarly, in our previous study, we also found that the expression of METTL3 and YTHDF2 was up‐regulated in bladder cancer and showed that METTL3 inhibited the expression of SETD7 and KLF4 in an m^6^A‐YTHDF2‐dependent manner to further promote the proliferation and metastasis of bladder cancer.^32^ In addition, a nine‐gene panel that included METTL3 was identified as a prognostic indicator for the recurrence of muscle invasive bladder cancer.^33^ Yang et al^34^ showed that METTL3 and ALKBH5 regulated m^6^A modification of the 3′‐UTR of the oncogene CDCP1 mRNA and that YTHDF1 recognized m^6^A modification to promote CDCP1 translation in bladder cancer. Similarly, Jin et al^35^ found that METTL3 and ALKBH5 regulated the m^6^A modification of the 3′‐UTR of the oncogene ITGA6 mRNA and that YTHDF1/3 recognized m^6^A modification to promote ITGA6 translation in bladder cancer. Recent studies have showed that METTL3 also regulates m^6^A modifications of non‐coding RNAs (miRNAs, lincRNAs and circRNAs).^48^, ^49^, ^50^ In bladder cancer, METTL3 regulates pri‐miR221/222 processing in an m^6^A‐dependent manner to promote carcinogenesis.^36^ In addition, a high expression pattern of METTL3 is demonstrated in bladder cancer via bioinformatic analysis.^37^ Therefore, METTL3 serves as an oncogene in the carcinogenesis of bladder cancer (Figure 2).

### 3.3 Renal cell carcinoma

**FIGURE 3:**
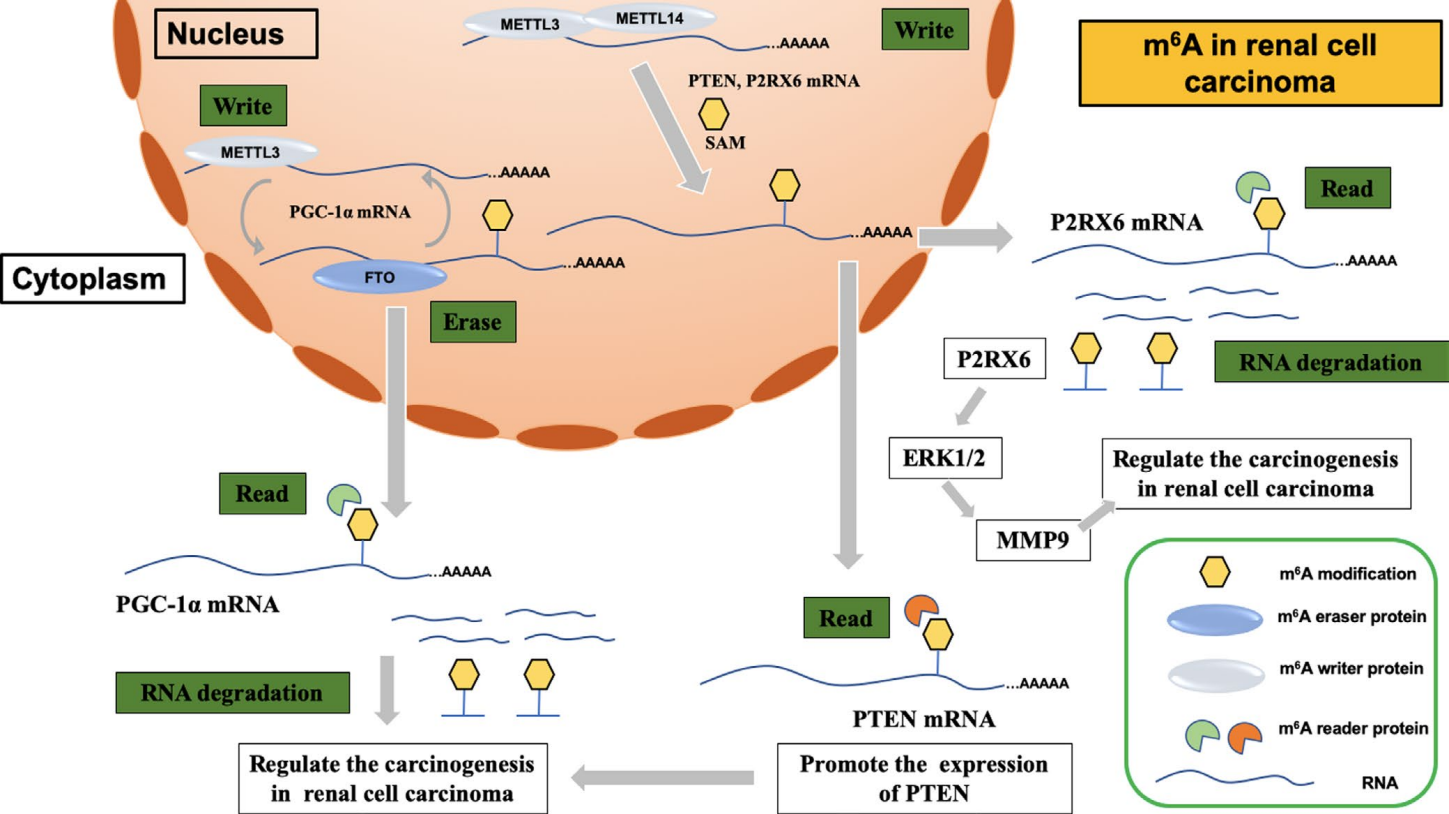
The possible mechanism of m^6^A methylation in renal cell carcinoma. METTL14 regulated PTEN and P2RX6 expression via m^6^A modification in renal cell carcinoma, and FTO suppressed carcinogenesis of renal cell carcinoma via the FTO‐PGC‐1α signalling axis

The incidence of renal cell carcinoma is still increasing rapidly worldwide.^51^ Although mortality is decreasing in developed countries, it is still a major problem in developing countries. Li et al^38^ found that the expression of METTL3 in renal cell carcinoma was down‐regulated and that the decreased expression of METTL3 was related to poor prognosis. They further elucidated that METTL3 inhibited proliferation and metastasis in renal cell carcinoma via epithelial‐to‐mesenchymal transition (EMT) and PI3K‐Akt‐mTOR pathways. In addition, down‐regulation of METTL14 in renal cell carcinoma was related to poor prognosis, and that METTL14 regulated PTEN expression via m^6^A modification, which indicated that METTL14 could possibly serve as a prognostic biomarker.^39^ A similar METTL14 expression pattern was found in renal cell carcinoma, and METTL14 further suppressed P2RX6 activation regulated metastasis of renal cell carcinoma via ATP‐induced Ca^2+^ influx modulating ERK1/2 phosphorylation and MMP9 signalling.^40^ Zhou et al^41^ further used the TCGA database to analyse the gene signatures and prognostic values of m^6^A regulators in renal cell carcinoma. They found that patients with any copy number variations (CNVs) of the m^6^A regulatory genes had worse overall survival (OS) and disease‐free survival (DFS) than those without CNVs; in addition, deletions of METTL3 and METTL14 were independent risk factors for poor OS. They also elucidated that decreased expression of METTL3 was related to activation of the mTOR pathway. Similar results were also observed by another team.^42^ They established a risk signature for predicting the prognosis of renal cell carcinoma based on METTL14 and METTL3. Interestingly, the down‐regulated expression of ALKBH5 and FTO was found to be related to poor overall survival and cancer‐specific survival in renal cell carcinoma, which implied that ALKBH5 and FTO could serve as potential prognostic biomarkers.^43^ In another study, the expression of FTO was also found to be down‐regulated in renal cell carcinoma, and FTO suppressed carcinogenesis of renal cell carcinoma via the FTO‐PGC‐1α signalling axis.^44^ To determine the potential downstream targets of m^6^A regulatory genes, m^6^A sequencing, RNA sequencing and bioinformatics analysis were used to demonstrate the first m^6^A transcriptome‐wide map of renal cell carcinoma and to identify differentially expressed mRNAs with m^6^A modifications.^52^ This could help to develop identify m^6^A‐related genes that can be exploited in new therapeutic strategies for renal cell carcinoma. The possible mechanism of m^6^A methylation in renal cell carcinoma is summarized in Figure 3.

## 4 DISCUSSION

### 4.1 Potential application of m^6^A modification in urological cancers

#### 4.1.1 m^6^A modifications as biomarkers

An increasing number of studies have indicated that m^6^A regulators could possibly be novel biomarkers in cancer diagnosis and prognostic evaluation. As an important m^6^A methyltransferase, METTL3 is well studied in many types of cancers. The expression of METTL3 is increased in gastric cancer and associated with poor prognosis, indicating that the expression of METTL3 is an independent prognostic factor and effective predictor in gastric cancer.^53^, ^54^ Similar results were also observed in hepatocellular carcinoma, pancreatic cancer, breast cancer, etc.^55^, ^56^, ^57^

As we mentioned before, METTL3 plays critical roles in both prostate cancer and bladder cancer, and the expression of METTL3 is elevated in these cancers. Taken together, considering the expression pattern of METTL3 in prostate cancer, bladder cancer and other cancers, and the role of METTL3 in the carcinogenesis of prostate cancer and bladder cancer, METTL3 could be a promising biomarker and prognostic indicator in prostate cancer and bladder cancer. However, unlike the expression pattern in prostate cancer, bladder cancer and most other types of tumours, METTL3 seems to be down‐regulated in renal cell carcinoma. Although its internal biological mechanisms need further elucidation, we found that METTL3 could possibly be used as a diagnostic biomarker and independent prognostic factor.

As crucial m^6^A‐binding proteins, the YTH domain family members play important roles in many types of cancers. The expression of YTHDF2 is up‐regulated in hepatocellular carcinoma, pancreatic cancer, etc,^55^, ^58^, ^59^ the expression of YTHDF1 is up‐regulated in colorectal cancer, hepatocellular carcinoma, ovarian cancer, lung cancer, etc,^60^, ^61^, ^62^, ^63^ and both YTHDF1 and YTHDF2 are independent risk factors for OS.

As we mentioned before, YTHDF1 and YTHDF2 play critical roles in both bladder cancer and prostate cancer. The expression of YTHDF1/2 is elevated in bladder cancer, and the expression of YTHDF2 is elevated in prostate cancer. Taken together, considering the expression pattern of YTHDF1/2 in prostate cancer, bladder cancer and other cancers, and the role of YTHDF1/2 in the carcinogenesis of prostate cancer and bladder cancer, YTHDF1/2 could be a promising biomarker and prognostic indicator in prostate cancer and bladder cancer.

#### 4.1.2 m^6^A modifications as therapeutic targets

The crucial roles of m^6^A modifications in urological cancers indicate that these modifications may become novel antitumour therapeutic targets. As crucial m^6^A regulators, METTL3 and YTHDF2 act as oncogenes in both prostate cancer and bladder cancer. The carcinogenesis of METTL3 and YTHDF2 in prostate cancer and bladder cancer mainly contributes to the inhibition of antitumour genes and the promotion of oncogenes, resulting in tumour development. Considering oncogenic role in prostate cancer and bladder cancer, METTL3 and YTHDF2 present opportunities for the development of effective targeted therapeutics. Small‐molecule METTL3 and YTHDF2 inhibitors could be designed and synthetised to examine the antitumour effects and safety in both prostate cancer and bladder cancer. Future studies could highlight the broad potential of targeting METTL3 and YTHDF2 for both prostate cancer and bladder cancer. In contrast, both m^6^A methyltransferases (METTL3 and METTL14) and m^6^A demethylases (FTO) act as tumour suppressor genes in renal cell carcinoma, indicating that the recruited m^6^A‐binding protein and potential downstream target play important roles in tumour development. However, in our ongoing research, we found that the m^6^A methyltransferase WTAP and the m^6^A demethylase ALKBH5 act as oncogenes in renal cell carcinoma. Therefore, due to the pathological diversity of renal cell carcinoma, the actual role of m^6^A regulators in it and its subtypes needs further elucidation.

PD‐1/PD‐L1‐related immunotherapy has proven to be effective in many types of tumours.^64^ Recent studies illustrated that PD‐1/PD‐L1‐related immunotherapy was effective in urological cancers.^65^, ^66^, ^67^, ^68^ FTO is demonstrated to promote carcinogenesis and anti‐PD‐1 resistance in melanoma, suggesting that FTO could be a potential therapeutic target in immunotherapy,^69^ and the deletion of YTHDF1 enhances the therapeutic efficacy of PD‐L1 checkpoint blockade, indicating that the m^6^A‐binding protein YTHDF1 could be another therapeutic target in antitumour immunotherapy.^70^ In addition, Wang et al^71^ identified the function of the m^6^A methyltransferase METTL3 in increasing the translation of immune transcripts.

As we mentioned before, m^6^A modification regulators, including METTL3, FTO and YTHDF1, play important roles in the carcinogenesis of urological cancers, suggesting that m^6^A modifications may be potential therapeutic targets for immunotherapy.

Chemotherapy and radiotherapy are commonly used in bladder cancer and prostate cancer, respectively. It is important to identify the chemoradiation sensitivity of each patient via certain indicators, and m^6^A regulators could be such indicators. METTL3 was found to increase the sensitivity of cells to anticancer reagents such as gemcitabine, 5‐fluorouracil, cisplatin and irradiation in pancreatic cancer, indicating the potential role of METTL3 in chemo‐ and radiotherapy resistance.^72^ In addition, FTO was found to enhance chemoradiotherapy resistance in cervical squamous cell carcinoma via β‐catenin.^73^

As we mentioned before, both METTL3 and FTO play crucial roles in carcinogenesis in both bladder cancer and renal cell carcinoma. Taken together, these findings suggest that m^6^A regulators could be potential therapeutic targets for patients receiving chemo‐ and radiotherapy.

#### 4.1.3 miRNAs and m^6^A modifications

In addition to the functions of m^6^A modifications in mRNA, recent studies have shown that m^6^A modifications also have roles in regulating non‐coding RNAs, especially microRNAs (miRNAs).^24^, ^48^, ^49^, ^50^ miRNAs are mainly processed by the microprocessor complex, which includes RNA‐binding protein DGCR8 and ribonuclease type III DROSHA.^74^ Alarcón et al^48^ found that METTL3 methylated pri‐miRNAs, facilitating their recognition and processing by DGCR8, indicating that the m^6^A regulator could possibly be a key factor in the initiation of miRNA biogenesis. They further identified that HNRNPA2B1 bound to m^6^A‐modified sites in a group of pri‐miRNAs, interacted with DGCR8, and promoted the maturation of pri‐miRNAs.^24^ In addition, METTL14 was identified to interact with DGCR8 and regulate the pri‐miR‐126 mature process in an m^6^A‐dependent manner in hepatocellular carcinoma.^75^ Pri‐miR‐25 in pancreatic cancer could be matured by smoking via enhanced m^6^A modification, which was catalysed by METTL3.^76^ A similar mechanism was found in bladder cancer. METTL3 interacted with DGCR8 and regulated the pri‐miR‐221/222 maturation process.^36^

Taken together, considering the crucial roles of m^6^A regulators and miRNAs in urological cancers and the potential regulatory mechanisms between m^6^A regulators and miRNAs, miRNAs and microprocessor proteins (such as DGCR8) could be potential therapeutic targets in urological cancers. However, future studies are needed to further clarify the underlying mechanisms.

#### 4.1.4 Alternative splicing and m^6^A modifications

Alternative splicing is the process of generating numerous mRNA variants from a single gene transcript, leading to proteome complexity and diversification.^77^ Alternative splicing exists in almost 95% of human genes and exerts functions in many biological aspects, including chromatin modification, signal transduction and carcinogenesis.^78^ In addition to cis‐regulatory elements, trans‐acting splicing factors including m^6^A regulators play critical roles in the alternative splicing process.^9^, ^79^ Previous studies indicated that dysregulation of m^6^A regulators drastically affects the process of alternative splicing. METTL3 regulated MyD88 alternative splicing on the lipopolysaccharide‐induced inflammatory response.^80^ FTO is involved in the alternative splicing process by triggering the inclusion of alternatively spliced exons and regulating the expression of terminal exons.^81^ HNRNPG interacts with RNA polymerase II to coregulate alternative splicing in m^6^A‐enriched exonic regions.^82^ HNRNPA2B1 phenocopies the effect of METTL3 to play an important role in alternative splicing on pri‐miRNA processing.^24^ YTHDC1 interacts with alternative splicing factors SRSF3 and SRSF10, and YTHDC1/SRSF3, and SRSF10 regulates splicing in an m^6^A‐dependent manner.^83^ In prostate cancer, YTHDC1 regulates CD44 alternative splicing, which is associated with carcinogenesis.^84^

Previous studies illustrated that alternative splicing plays important roles in urological cancers. In prostate cancer, androgen receptor spliced variants (AR‐Vs) have been implicated in the carcinogenesis of metastatic prostate cancer, which contribute to resistance to both anti‐androgen therapy and radiotherapy.^85^ In bladder cancer, Xie et al^86^ illustrated that PTBP1 promotes bladder cancer lymph node metastasis and cell proliferation via an alternative splicing dependent mechanism and that PTBP1 could be a novel prognostic marker and therapeutic target. In renal cell carcinoma, SF3B3 modulates EZH2 alternative splicing to promote carcinogenesis, and SF3B3 could be a potential prognostic factor and therapeutic target in renal cell carcinoma.^87^

Therefore, understanding the role of the m^6^A‐regulated alternative splicing in the carcinogenesis of urological cancers and exploring the therapeutic application of manipulating alternative splicing to improve urological cancer patient care are of great importance in the future.

### 4.2 Brief summary

The regulatory functions of m^6^A modifications include RNA degradation, translation, processing and splicing. Although m^6^A modifications are also involved in the carcinogenesis of many types of cancers, including urological cancers, the potential mechanisms should be further studied. METTL3 has different expression patterns in different urological cancers. Considering the dual role of METTL3 as an oncogene in prostate cancer and bladder cancer and as a tumour suppressor gene in renal cell carcinoma, its underlying mechanisms need to be further studied. In addition, the mechanisms by which m^6^A‐binding proteins co‐ordinate their different functions in certain types of cancers need to be further elucidated. Additionally, some m^6^A modification–based enzyme inhibitors have demonstrated potential effects on cancer; however, there is still a long way to go before m^6^A‐based cancer therapy can be applied in the clinic.^88^

## 5 CONCLUSIONS

Epigenetics has long been a hot topic in the field of scientific research.^89^ RNA m^6^A modifications are of great importance in urological cancers and serve as diagnostic and prognostic biomarkers that regulate carcinogenesis and metastasis, indicating their potential as therapeutic targets. However, further studies are still necessary to elucidate the underlying mechanisms in urological cancers so that these findings can be translated from bench to bedside in the future.

## CONFLICT OF INTEREST

The authors confirm that there are no conflicts of interest.

## AUTHOR CONTRIBUTION

**Xiao Wang:** Writing‐original draft (lead). **Haiyun Xie:** Data curation (equal). **Yufan Ying:** Data curation (equal). **Danni Chen:** Writing‐review & editing (lead). **Jiangfeng Li:** Project administration (lead).
